# Trends in the sample size, statistics, and contributions to the BrainMap database of activation likelihood estimation meta‐analyses: An empirical study of 10‐year data

**DOI:** 10.1002/hbm.26177

**Published:** 2022-12-08

**Authors:** Andy Wai Kan Yeung, Michaela Robertson, Angela Uecker, Peter T. Fox, Simon B. Eickhoff

**Affiliations:** ^1^ Oral and Maxillofacial Radiology Applied Oral Sciences and Community Dental Care, Faculty of Dentistry, The University of Hong Kong Hong Kong China; ^2^ Research Imaging Institute University of Texas Health Science Center San Antonio Texas USA; ^3^ Department of Radiology University of Texas Health Science Center San Antonio Texas USA; ^4^ Institute of Systems Neuroscience, Medical Faculty Heinrich Heine University Düsseldorf Düsseldorf Germany; ^5^ Institute of Neuroscience and Medicine, Brain & Behaviour (INM‐7) Research Centre Jülich Jülich Germany

**Keywords:** activation likelihood estimation, fMRI, meta‐analysis, neuroimaging, reproducibility, statistical threshold

## Abstract

The literature of neuroimaging meta‐analysis has been thriving for over a decade. A majority of them were coordinate‐based meta‐analyses, particularly the activation likelihood estimation (ALE) approach. A meta‐evaluation of these meta‐analyses was performed to qualitatively evaluate their design and reporting standards. The publications listed from the BrainMap website were screened. Six hundred and three ALE papers published during 2010–2019 were included and analysed. For reporting standards, most of the ALE papers reported their total number of Papers involved and mentioned the inclusion/exclusion criteria on Paper selection. However, most papers did not describe how data redundancy was avoided when multiple related Experiments were reported within one paper. The most prevalent repeated‐measures correction methods were voxel‐level FDR (54.4%) and cluster‐level FWE (33.8%), with the latter quickly replacing the former since 2016. For study characteristics, sample size in terms of number of Papers included per ALE paper and number of Experiments per analysis seemed to be stable over the decade. One‐fifth of the surveyed ALE papers failed to meet the recommendation of having >17 Experiments per analysis. For data sharing, most of them did not provide input and output data. In conclusion, the field has matured well in terms of rising dominance of cluster‐level FWE correction, and slightly improved reporting on elimination of data redundancy and providing input data. The provision of Data and Code availability statements and flow chart of literature screening process, as well as data submission to BrainMap, should be more encouraged.

## Graphical abstract

- Cluster‐level FWE became the mainstream in recent years for ALE‐type CBMA.
- More ALE papers should exclude ROI papers and report details on data redundancy filter.
- They should provide Data/Code availability statements and flow chart of paper screening.

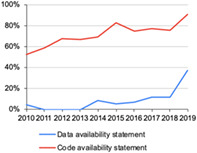

## 1 INTRODUCTION

Standardized, atlas‐referenced, three‐dimensional (3D; *x*–*y*–*z*) Cartesian coordinates for neuroimaging analysis and reporting were originally introduced with the explicit goal of “facilitating direct comparison of experimental results from different laboratories, even when different imaging techniques are employed” (Fox et al., 1985). This vision has been abundantly realized through the development and ongoing evolution of an extensive coordinate‐based meta‐analysis (CBMA) literature. The first CBMAs, published in the early 1990s (de Jong et al., 1994; Fox, 1995; Frith et al., 1991; Paus, 1996), were statistically informal: plotting coordinates to illustrate spatial convergence and computing between‐paper means and standard deviations for individual brain regions. Since then, several CBMA approaches have been developed and steadily refined to enhance statistical rigor. (For a review of the evolution of CBMA, readers are referred to [Fox et al., 1998; 2014]). As with all meta‐analyses, CBMA reduces a segment of the literature (often a very large segment) to its most salient and reproducible findings. Considering both the outsized impact of the neuroimaging literature on human neuroscience and the well‐publicized shortcomings of statistical rigor in this literature (Button et al., 2013; Eklund et al., 2016; Woo et al., 2014), it is timely to consider the CBMA literature from the standpoint of best practices for rigor, reproducibility and transparency. The overall goal of the analyses reported here was to assess compliance of this important literature segment with available best practice guidelines.

Open science that adheres to data analysis and publishing best practices are important for all fields. Transparency and reproducibility are particular key aspects of best practices that are being increasingly adopted by journals internationally (Iqbal et al., 2016; Munafò et al., 2017; Wallach et al., 2018). Positive community and publisher response to these principles have led to the increasingly common requirement that the Methods sections of articles have Data Availability and Code Availability statements (Rousi & Laakso, 2020). For CBMA, the provision of a list of all source papers that supplied the original data (coordinates) should be expected. Without such a list, it becomes impossible for readers to verify and replicate the meta‐analytic findings.

Flow chart is a reader‐friendly feature of research papers, and its usage is now widely adopted to clearly illustrate all data gathering and pruning procedures. Clinical trials best practices guidelines are specific about this, and such publications should follow the Consolidated Standards of Reporting Trials (CONSORT) guideline (Schulz et al., 2010) and include a CONSORT flow diagram. For meta‐analysis, following the Preferred Reporting Items for Systematic Reviews and Meta‐Analyses (PRISMA) guideline (Moher et al., 2009) and submitting a completed PRISMA checklist are now mandatory for many journals. The guideline also requires a PRISMA flow diagram showing data gathering (with data search statements on database selection and search terms) and all pruning rules and consequences. These flow charts should therefore support the principles of transparency and reproducibility and become a necessary part of research publications.

False positives, even under the open science framework, is considered as one crucial issue in neuroimaging caused by faulty correction for multiple comparisons, small sample size, and variability of analysis methods (Button et al., 2013; Eklund et al., 2016; Woo et al., 2014). Faulty correction for multiple comparisons is particularly problematic for cluster‐level inference, such that a liberal cluster‐defining primary threshold often increases false positives and produces large clusters that are anatomically uninformative (Woo et al., 2014). A software bug was also reported to reduce the size of the image searched for clusters and hence underestimate the severity of multiplicity correction (Cox et al., 2017; Eklund et al., 2016). Meanwhile, a small sample size leads to low statistical power and renders a significant result less likely to be reflecting a true effect (Button et al., 2013). To exacerbate the situation, the variability of analysis methods without clear reporting would render results irreproducible (Botvinik‐Nezer et al., 2020; Carp, 2012a). Encountering these difficulties, the neuroimaging community takes rigor, reproducibility and transparency very seriously. The Organization for Human Brain Mapping (OHBM) has established the Committee on Best Practices in Data Analysis and Sharing (COBIDAS), and already issued best practices documents for MRI (Nichols et al., 2017) and MEEG (Pernet et al., 2020), respectively. The OHBM also formed an Open Science Special Interest Group (SIG) to organize educational and promotional projects that raise the awareness of open science (Organization for Human Brain Mapping, 2021).

Advances in CBMA statistical rigor have been ongoing since 2002. One of the commonest CBMA approach in the literature is the activation likelihood estimation (ALE) approach (Acar et al., 2018; Samartsidis et al., 2020; Tahmasian et al., 2019; Yeung et al., 2019). It was introduced by Turkeltaub et al. (2002) and usually operated with the software GingerALE developed by the BrainMap project (Laird, Eickhoff, Kurth, et al., 2009). ALE is a mass‐univariate method for meta‐analytically computing likelihood of finding an activation (or atrophy) at any given brain voxel in a specific set of behavioural (task activation) or clinical (group‐wise contrast) conditions from the literature reporting results of similar contrasts in standardized coordinates. Another less common CBMA approach is the kernel density analysis (KDA), for which a significant result indicates that the density of peaks within *r* mm of a voxel is greater than that can be expected by chance (Wager et al., 2007). There is also an approach called Seed‐based d mapping (formerly signed differential mapping, SDM), which combines data from effect size, reported coordinates and statistical parametric maps (Albajes‐Eizagirre et al., 2019). Regardless of the approach, researchers can search for relevant papers indexed in BrainMap database through a guided user interface, export the selected experimental results for analyses, and even input unindexed papers and data into the database (Fox et al., 2005; Fox & Lancaster, 2002; Laird et al., 2005; Vanasse et al., 2018). Searching BrainMap database should be more convenient than searching other literature databases with the following merits: (1) BrainMap indexes brain imaging experiments only; (2) it provides very well‐curated and cleaned data sets checked by internal coders; (3) ROI‐based analyses are excluded and (4) The coordinate system and analytic software used to generate the data are checked and thus the best available transforms between systems are provided. Meanwhile, PRISMA criteria expect a comprehensive search of the literature including the recently published papers (Moher et al., 2009), so that searching other literature databases may complement the search results to ensure recency. On a separate note, there are other popular analyses that rely entirely on BrainMap data on a 100% basis, such as meta‐analytic connectivity modelling (MACM) (Laird, Eickhoff, Li, et al., 2009; Robinson et al., 2010), co‐activation based parcellation (CBP) (Bludau et al., 2014), behavioural decoding with behavioural domains and disease decoding with ICD‐10 codes (Fox & Lancaster, 2002; Laird, Eickhoff, Kurth, et al., 2009; Lancaster et al., 2012).

Compliance of the ALE literature with available best practice guidelines is crucial, the meta‐analysis is itself a study requiring careful planning and execution (Jones, 1995). Based on recent guidelines specifically for CBMA (Müller et al., 2018; Tahmasian et al., 2019), we selected a sub‐set that best reflects the main aspects of ALE publications to be evaluated: reporting standards, study characteristics, statistical threshold and data sharing.

## 2 MATERIALS AND METHODS

### 2.1 Database and selection criteria

All publications listed in the BrainMap Publications page (https://www.brainmap.org/pubs/) were initially included. Based on the list, papers published from 2010 until 2019 were screened (*n* = 867). We queried the PubMed database by the searching “activation likelihood estimation,” “coordinate‐based meta‐analysis,” or “ALE meta‐analysis,” and returned with 568 papers. It implied that the list from BrainMap page is reflective of the actual literature without missing many papers. Papers were excluded with the following reasons: (1) no ALE meta‐analysis results (*n* = 254). These excluded papers mainly dealt with multi‐variate modelling only (such as MACM, CBP, independent component analysis [ICA], and graph theory modelling [GTM]) instead of ALE; (2) not journal publications (*n* = 5, among which one was a preprint of another included paper and one was a duplicate entry); (3) not retrievable (*n* = 2) and (4) not written in English (*n* = 3). Finally, 603 publications reporting ALE meta‐analyses were included for the subsequent evaluation.

### 2.2 Data extraction

All five authors designed a data extraction sheet together. One author (AWKY) extracted data from the 603 publications to be double checked by another author (SBE). Internal data from the BrainMap database was provided by two authors (MR and AU). For each publication, the following data were extracted:

1. Publication year.
2. Journal impact factor (in their respective publication year).
3. Total number of source papers and a list of all of them (yes/no).
4. Data Availability and Code Availability statements anywhere in the text (yes/no). The provision of these information supports open science and facilitates study replication (Poldrack & Gorgolewski, 2014). In this item, data means the coordinates from the source papers. Code Availability statement was coded “yes” if the used software was stated and available online (e.g., GingerALE) with the used version reported, or if the used in‐house code was available online.
5. Use of a flow chart (PRISMA diagram) and provision of data searching statements (i.e., databases and search terms) to illustrate the data screening and pruning process (yes/no).
6. Statement of inclusion/exclusion criteria (yes/no).
7. Explicit exclusion of papers reporting ROI results only (yes/no). ROI papers should be excluded as they create bias in spatial convergence (Eickhoff et al., 2012; Müller et al., 2018; Turkeltaub et al., 2002).
8. Number of ALE meta‐analysis conducted, counting only main effects.
9. Correction of statistical inference for multiple comparisons (cluster‐level, voxel‐level or unknown). Cluster‐level inference should enable a more optimized result.
10. Choice of most stringent statistical correction (FWE, FDR, uncorrected, threshold‐free cluster enhancement [TFCE] or unknown). Voxel‐wise FDR correction is nowadays considered unsuitable for ALE or even neuroimaging data due to high false positive rates (Chumbley et al., 2010; Chumbley & Friston, 2009; Eickhoff et al., 2016).
11. Average number of Experiments in each analysis. Having few Experiments per analysis may cause underpower.
12. Number of papers in each analysis (applicable only if number of Experiments was unclear).
13. Data redundancy filter (merge multiple Experiments into one, select the most representative Experiment or unknown). Treating multiple dependent datasets as independent may exaggerate the influence of the concerned subjects and bias the results (Turkeltaub et al., 2012).
14. Double‐checking with the original sources for the extracted coordinates.
15. Contribution to the BrainMap database (“Complete Citation” including links to the Sleuth and GingerALE datasets, used Scribe, provided BrainMap workspace, or none).
16. Dataset availability as supplementary files or links to repository (input file .txt, output file .nii, both or neither).
17. BrainMap as one of the searched databases for Paper identification (yes/no).

For point (15), it is likely that many contributors/authors may code and submit data to the BrainMap database without explicitly mentioning it in their paper. Therefore, we also obtained the internal statistics from the BrainMap team to see how many source papers were coded by internal and external contributors respectively during the study period (2010–2019). This should provide a more balanced account of the contributions by the community to the BrainMap database.

### 2.3 Statistical analysis

Statistical analyses were conducted in SPSS 26.0 (IBM, NY). Linear regressions were conducted to evaluate if linear trends existed for the annual number of ALE papers published and journal impact factor. Then, tests were conducted to evaluate three aspects: reporting standards, study characteristics, and data sharing. For reporting standards, binomial logistic regressions were conducted to evaluate if publication year was an influencing factor on the ratio of papers that provided a list of all source papers, Data and Code availability statements, flow chart that illustrated the literature screening process, data searching statements, the inclusion/exclusion criteria, and explicit exclusion of ROI papers. Multinomial logistic regressions were conducted to evaluate if publication year was an influencing factor on the choice of statistical threshold, and strategy to adjust for multiple related Experiments from the same set of subjects.

For study characteristics, linear regressions were conducted to evaluate if linear trends existed for the number of Papers (source papers) in each ALE paper, number of ALE meta‐analysis per paper and number of Experiments per analysis. Binomial logistic regression was conducted to evaluate if publication year was an influencing factor on the ratio of papers searching of BrainMap database as one of their data sources.

For data sharing, multinomial logistic regressions were conducted to evaluate if there were trends in the contributions to BrainMap database, and data availability (provision of input and output data).

We reasoned that papers that supplied data to BrainMap database (provided “Complete Citation” or brain coordinates not indexed in the database) might welcome others to examine their data and thus their reporting standard and study characteristics could potentially be of higher standard. Hence, papers were then divided into two groups, those with contributions to BrainMap database and those without. Independent sample *t‐*tests were conducted to evaluate if these groups differed in terms of mean number of source papers analysed, journal impact factor, number of ALE meta‐analyses, and number of Experiments per analysis. Chi‐squared tests were conducted to evaluate if these groups differed in the ratio of stated inclusion/exclusion criteria, explicit exclusion of ROI papers, data redundancy filter, data availability and statistical threshold.

Reporting standards between well‐powered and less‐powered papers might also be different, because well‐powered papers could spare/exclude unsuitable data and adopt a higher reporting standard. Therefore, papers were first divided into two groups by median splitting their number of source papers analysed. Better‐powered studies (*n* = 283) had >28 source papers analysed, whereas less‐powered studies (*n* = 318) had 28 or fewer source papers analysed. Chi‐squared tests were used to evaluate if the two groups differed in terms of ratio of adjusting for multiple related Experiments, explicit exclusion of ROI papers, and use of cluster‐level FWE. The comparisons were then repeated by splitting the papers into those having a mean of >17 Experiments per analysis (*n* = 126) against those <17 (*n* = 183; the rest 294 papers did not report number of Experiments per analysis).

Results of all statistical tests were significant if *p* < .05. This study did not involve human or animal subjects, hence ethical approval was not applicable.

## 3 RESULTS

**FIGURE 1:**
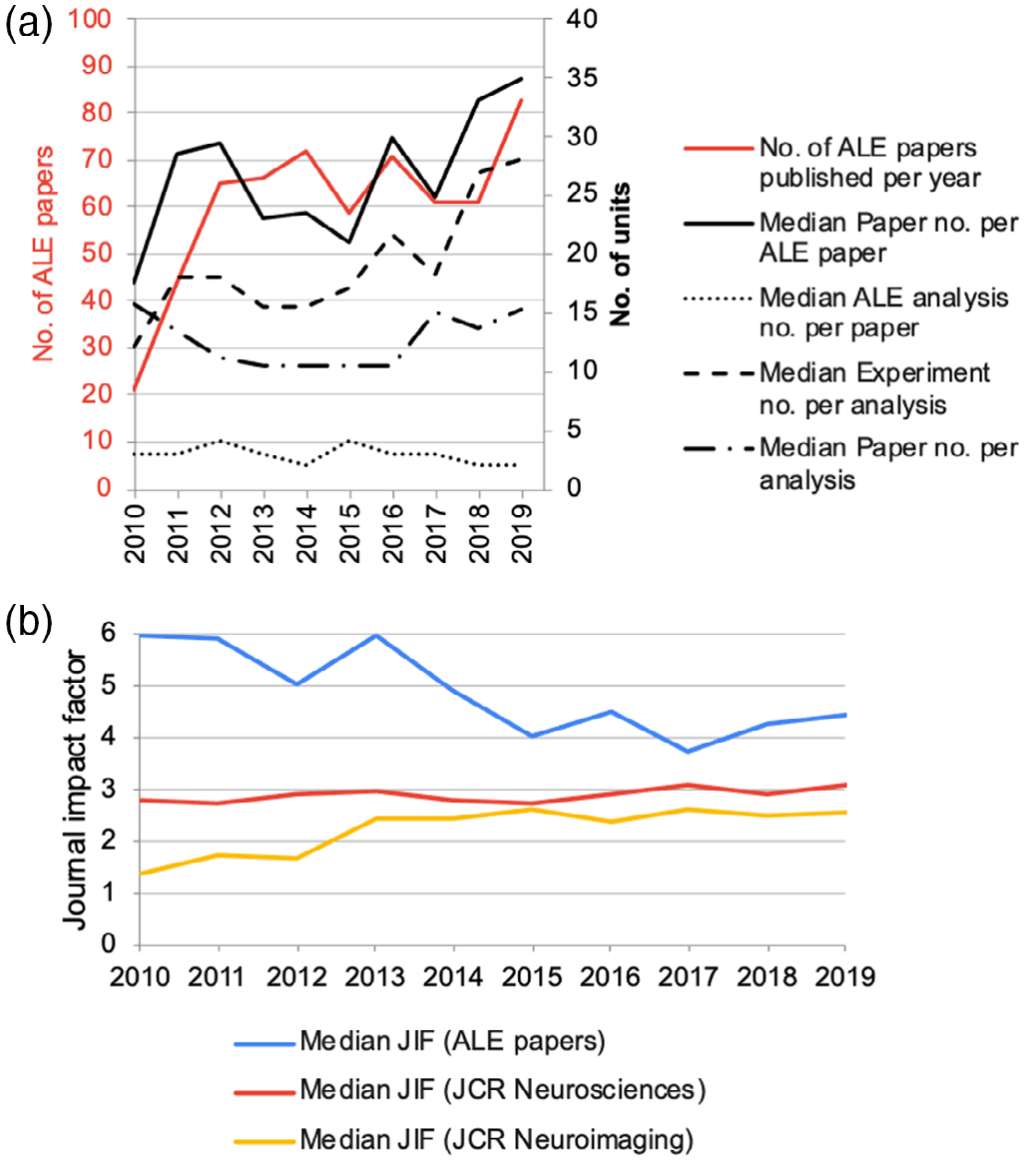
Evolution of the ALE papers over the 2010s. (a) The number of ALE papers published per year and their characteristics regarding sample sizes. (b) The median journal impact factor in each year.

The detailed descriptive data is listed in Table 1. The data extraction sheet with coded data is available at Figshare (https://doi.org/10.6084/m9.figshare.21580998). The number of ALE papers published per year rose sharply during the first half of the decade (Figure 1a), and in overall showed an increasing trend (*p* = .022, Table 2). At the same time, journal impact factor of the papers showed a decreasing trend (*p* = .001, Figure 1b, Table 2). Compared with the median impact factor of Neurosciences and Neuroimaging journals, it seemed that the impact of ALE papers was higher than the average papers. Findings regarding reporting standards, study characteristics, and data sharing are reported in below sections.

**TABLE 1:** Overall characteristics of the 603 surveyed ALE publications

Parameter	Value
Journal impact factor	Mean = 4.93, median = 4.36, SD = 3.13, range = 0–33.65
Open science	Data Availability statement, *n* = 60 (10.0%) Code Availability statement, *n* = 445 (73.8%) Data Searching statement, *n* = 562 (93.2%) Flow Chart of literature screening process, *n* = 199 (33.0%) List of Source Papers, *n* = 575 (95.4%)
Total number of source papers (Papers) involved in an ALE publication	Mean = 49.6, median = 28.0, SD = 80.5, range = 4–811 (2 papers did not report this)
No. of ALE meta‐analysis conducted and reported	Mean = 4.2, median = 3.0, SD = 4.2, range = 1–37
Level of inference	Voxel‐level, *n* = 377 (62.5%) Cluster‐level, *n* = 218 (36.2%) Unclear, *n* = 8 (1.3%)
Correction for multiple comparison	FDR, *n* = 339 (56.2%) FWE, *n* = 213 (35.3%) Uncorrected, *n* = 28 (4.6%) TFCE, *n* = 1 (0.2%) Unclear, *n* = 22 (3.6%)
No. of Experiments (known as Contrasts in some papers) in each meta‐analysis, *n* = 309 (51.2%)	Mean = 42.0, median = 20.6, SD = 121.4, range = 2.6–1827.0
No. of Papers in each meta‐analysis (only applicable to those not reporting number of Experiments), *n* = 238 (39.5%)	Mean = 19.3, median = 12.5, SD = 22.5, range = 3.3–203.0
Adjustment for data redundancy	Unclear, *n* = 528 (87.6%) Selected the most representative Experiment, *n* = 56 (9.3%) Merged multiple Experiments into one, *n* = 19 (3.2%)
Contribution to BrainMap database	No contribution, *n* = 552 (91.5%) “Complete Citation” including links to Sleuth and GingerALE datasets, *n* = 36 (6.0%) Used Scribe, *n* = 14 (2.3%) Provided BrainMap workspace file, *n* = 1 (0.2%)
Data availability	None, *n* = 565 (93.7%) Input files, *n* = 19 (3.2%) Output files, *n* = 17 (2.8%) Both files, *n* = 2 (0.3%)
Use of BrainMap database as data source	Not reported, *n* = 526 (87.2%) Yes, *n* = 77 (12.8%)

**TABLE 2:** Trends in the parameters over the decade

Test	Parameter	*p* value	Trend
Linear regression	No. of ALE papers published	.022	Increase
	Journal impact factor	.001	Decrease
	No. of Papers included per ALE paper	.360	n.s.
	No. of meta‐analyses per ALE paper	.031	Decrease
	No. of Experiments per analysis	.457	n.s.
	No. of Papers per analysis	.148	n.s.
Binomial logistic regression	Data availability statement	<.001	Increase
	Code availability statement	<.001	Increase
	Data searching statements	<.001	Increase
	Flow chart of literature screening	<.001	Increase
	List of all source papers	.702	n.s.
	Statement of inclusion/exclusion criteria	.933	n.s.
	Explicit exclusion of ROI papers	.173	n.s.
	BrainMap database as a data source	.036	Increase
Multinomial logistic regression	Most stringent statistical threshold chosen^a^	<.001	Cluster‐level FWE: increase Voxel‐level FDR: decrease Voxel‐level uncorrected: increase
	Adjustment for multiple related Experiments^b^	.025	Merge into one: increase Select the most presentative: n.s.
	Contribution to BrainMap^c^	<.001	Provide Complete Citation: decrease Use Scribe or provide Workspace file: n.s.
	Data availability^d^	<.001	Provide input file: increase Provide output file: n.s. Provide both: n.s.

Abbreviation: n.s., not significant.

^a^ The prevalence was compared against the reference category: others.

^b^ The prevalence was compared against the reference category: unknown.

^c^ The prevalence was compared against the reference category: no contribution.

^d^ The prevalence was compared against the reference category: provide neither file.

### 3.1 Reporting standards

**FIGURE 2:**
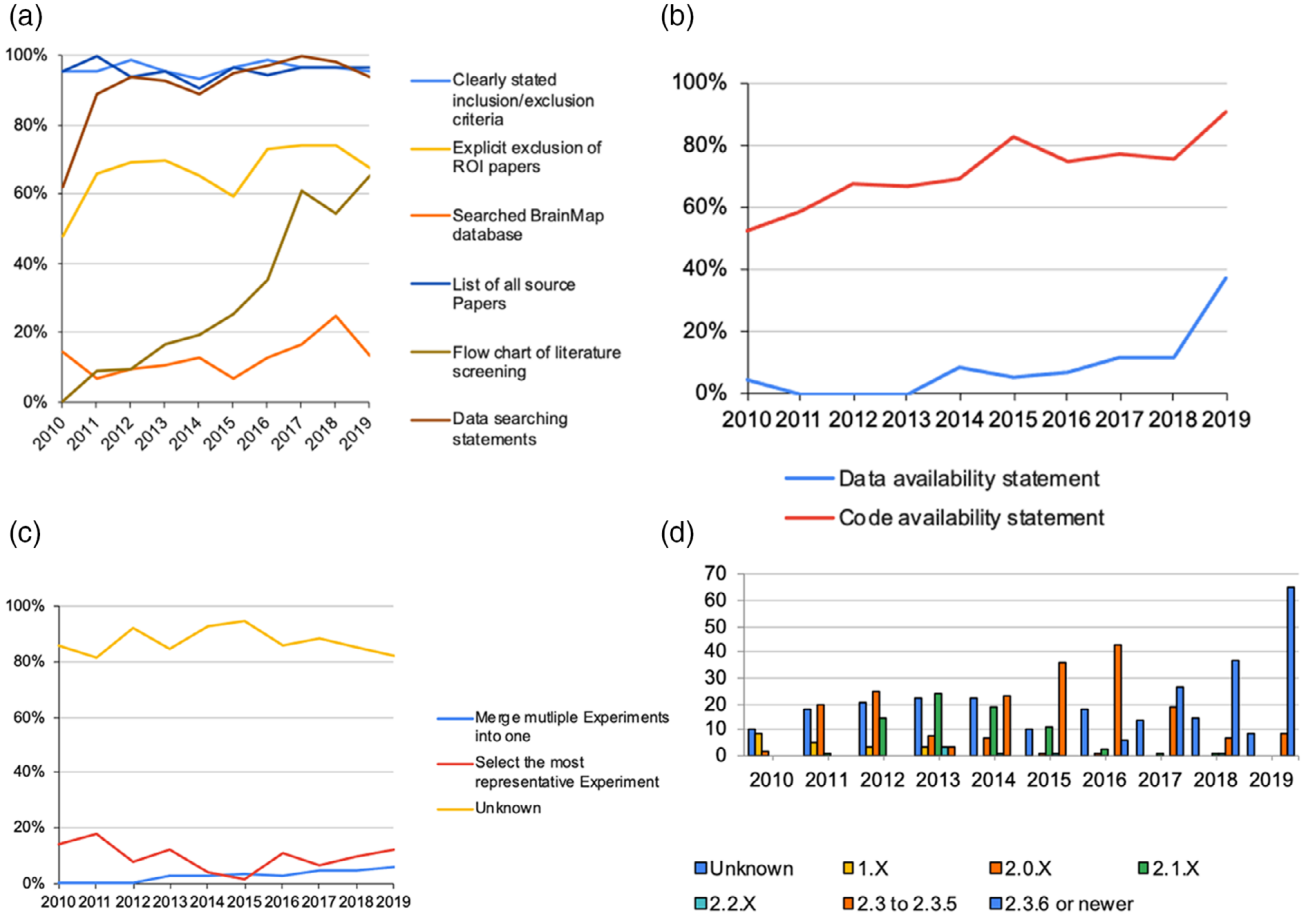
Open science and reporting transparency of the ALE papers over the 2010s. (a) The ratio of ALE papers reporting data searching statements, selection criteria, explicit exclusion of ROI papers, flow chart of literature screening process, list of all source papers, and searching of BrainMap database as one of their sources of data. (b) The provision of Data and Code availability statements. (c) The strategy to adjust for multiple related Experiments from the same set of subjects. (d) Versions of GingerALE used.

Most ALE papers had data searching statements (93.2%), a list of all source papers (95.4%), and inclusion/exclusion criteria on Paper selection (96.2%). Meanwhile, only 68.0% of the papers explicitly excluded ROI papers, with the annual ratio always exceeding 65% since 2011 (except in 2015 with 59.3%, Figure 2a) with no significant overall trend (*p* = .173). The use of flow chart and Data availability statement were scarce (33.0% and 10.0%, respectively), whereas Code availability statement was common (73.8%). Their usages showed an increasing trend (Figure 2a,b, Table 2).

### 3.2 Statistical inference and multiple comparisons correction

**FIGURE 3:**
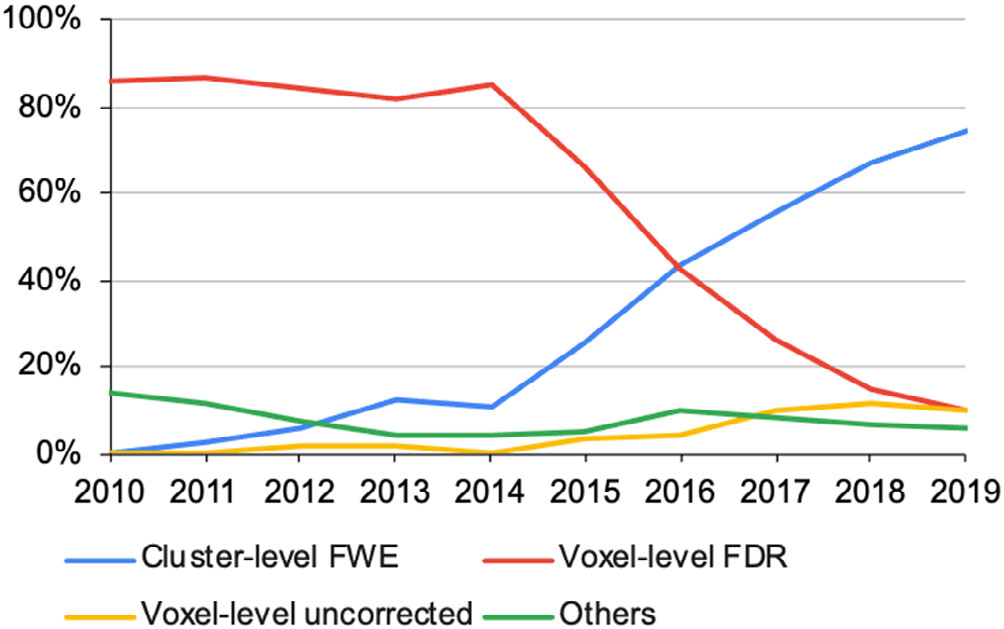
The choice of statistical thresholds of the ALE papers.

Considering level of inference and correction method together, the most prevalent statistical thresholds were voxel‐level FDR (54.4%) and cluster‐level FWE (33.8%). Voxel‐level FDR has been the predominant choice in the early 2010 s accounting for >80% of papers per year (Figure 3). Since 2016, voxel‐FDR has fallen out of favour and its share was largely taken up by cluster‐level FWE and, by a much smaller extent, voxel‐level uncorrected (although total n is very small for this, *n* = 28). This change corresponds to (Eickhoff et al., 2016) that recommended against FDR and for FWE. The voxel‐level uncorrected papers often investigated uncommon disease/population groups, such as DRD2 rs1076560 polymorphism (risk factor for schizophrenia) (Luykx et al., 2017), children with developmental coordination disorder (Fuelscher et al., 2018), and patients with Tourette Syndrome (Polyanska et al., 2017). Across the whole decade, the prevalence of voxel‐level FDR had a decreasing trend whereas cluster‐level FWE and voxel‐level uncorrected had an increasing trend (*p* < .001, Table 2).

Most papers did not clearly report on data redundancy filter (87.6%). For those paper that mentioned this, selecting the most representative Experiment was more common than merging multiple Experiments into one (Figure 2c). The former had an increasing trend over the years whereas the latter did not (Table 2). Still, both methods together only accounted for <20% of ALE papers published in each year. The rest did not state how to manage this issue or if they enter the same group of subjects multiple times.

Most papers did not explicitly mention that they double‐checked with the original sources about the accuracy of the coordinates to be entered into the meta‐analysis (90.7%).

### 3.3 Study characteristics

Most ALE papers reported the total number of Papers involved (99.7%), with no significant trend over the survey period (*p* = .360, Figure 1a, Table 2). Each paper had an average of 4.2 ALE meta‐analyses, and this number showed a negative trend (*p* = .031). Each ALE analysis had an average of 42.0 Experiments, which showed no significant trend (*p* = .457). About one‐third (30.3%, *n* = 183) of the papers had an average of >17 Experiments per analysis as recommended.

Better‐powered studies (total number of original studies >28) were more likely to use cluster‐level FWE than less‐powered studies (40.6% vs. 27.7%, *p* < .001). They were also more likely to apply data redundancy filter (16.3% vs. 9.1%, *p* = .024). The two groups did not differ in terms of explicit exclusion of ROI papers (67.0% vs. 68.9%, *p* = .614). If we only considered papers reporting number of Experiments per analysis, then better‐powered studies (average number of Experiments per analysis >17) were more likely to use cluster‐level FWE than less‐powered studies (50.8% vs. 29.4%, *p* < .001). They did not differ in terms of adjusting for multiple related Experiments (7.1% vs. 3.2%, *p* = .175) or explicit exclusion of ROI papers (76.5% vs. 73.0%, *p* = .486).

As additional information, most papers did not report searching the BrainMap database as one of their data sources (87.2%), but there was an overall increasing trend in its searching (*p* = .036, Figure 2a, Table 2). Meanwhile, 73.6% of the papers mentioned the version of GingerALE used, with another 3.2% using an in‐house build of it. The successions of the versions are illustrated in Figure 2d. Since 2017, the predominant types were version 2.3.6 or newer variants, which did not contain bugs embedded in older versions that would inflate false positive results (Eickhoff et al., 2017).

### 3.4 Data sharing

**FIGURE 4:**
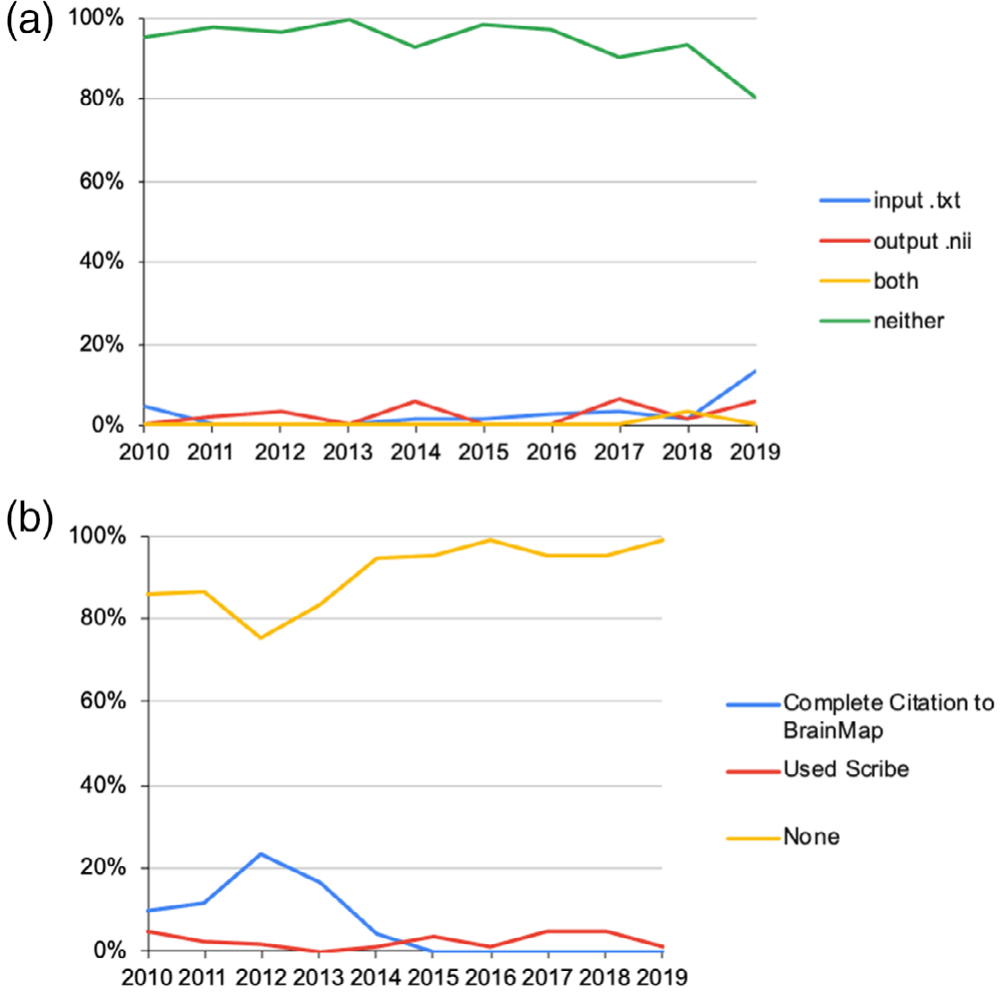
Relationship with BrainMap products. (a) Data availability. (b) The contributions to BrainMap database.

Most papers did not provide input file for and output ALE map from GingerALE (93.7%, including two papers providing broken links). Over the year, the ratio of ALE papers providing neither input data file (in .txt or any other text format for GingerALE) nor output file always exceeded 90%, except in 2019 the ratio dropped to 80.7% (Figure 4a). The ratio of papers providing the input files showed an increasing trend (Table 2).

**FIGURE 5:**
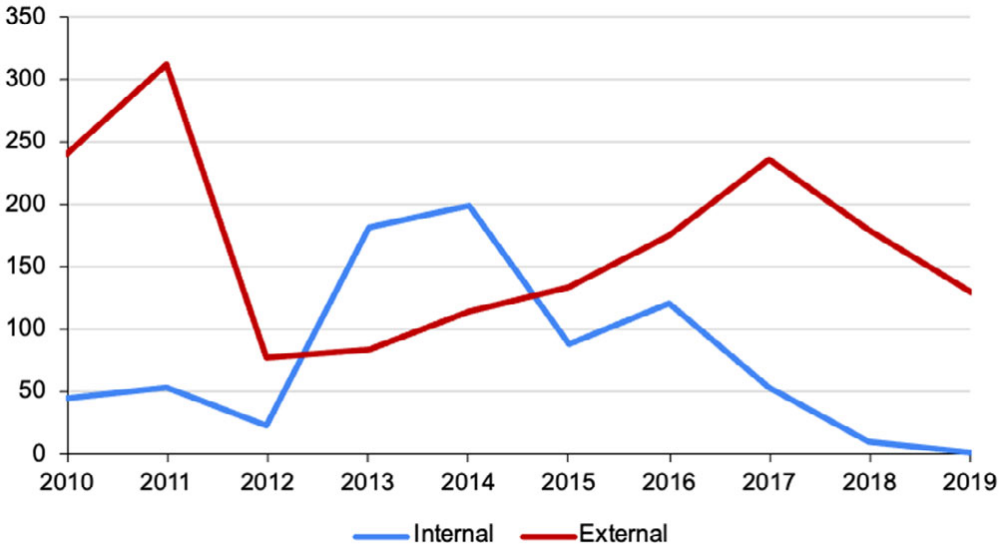
Number of source papers coded and submitted to BrainMap database by internal and external contributors during 2010–2019. A total of 61% (2928 out of 4826) was inputted by external contributors.

Most papers did not explicitly mention that they contributed to the BrainMap database (91.5%). In other words, they did not provide “Complete Citation” or use Scribe. The ratio of having contributions never exceeded 25% in a single year (Figure 4b), and there was a decreasing trend in providing “Complete Citation” but no significant trend in using Scribe (Table 2). Some papers might have supplied their output files to online repositories or databases without an explicit statement in the main text or publisher's website, so these might be missed. In fact, statistics obtained from the BrainMap team showed that external contributors coded 61% of the 4826 source papers added into the BrainMap database during the period of 2010–2019 (Figure 5). This implied that the neuroimaging community actually largely supported and contributed to the content of the BrainMap project.

Papers with contributions to BrainMap database had a significantly lower mean number of source papers analysed (25.9, SD = 22.4) than their counterparts (51.8, SD = 83.6; *p* < .001). They did not significantly differ in terms of journal impact factor, number of ALE meta‐analyses, number of Experiments per analysis, ratio of stated inclusion/exclusion criteria, explicit exclusion of ROI papers, data redundancy filter, data availability and statistical threshold (*p* > .05).

## 4 DISCUSSION

Trends and shortcomings are discussed below. A specific recommendation was made for every shortcoming, and these recommendations are listed in Box 1.

BOX 1 **Recommendations for conducting a CBMA meta‐analysis**

- State Data Availability and Code Availability in the Methods section or per journal policy.
- Provide PRISMA‐style flowchart and prose descriptions of inclusion and exclusion criteria.
- Search BrainMap database first (Scribe). Conduct exhaustive search second.
- State CBMA standards compliance of input data: whole‐brain, voxel‐wise analyses; comprehensive coordinate reporting; exclusion of ROI studies.
- State CBMA analysis choices: correction for multiple comparisons; data‐redundancy filter method, and so on.
- Code (Scribe) and submit (BrainMap.org) all papers not already in BrainMap. State this in Data Availability.
- Provide supplementary materials with your journal submission: spreadsheet of input papers; BrainMap workspace; GingerALE input file; nifty (.nii) output file.
- Contribute your paper and supplementary materials to BrainMap.org/pubs after acceptance, to create a “complete citation.”
- Contribute output file (.nii) to ANIMA database.

### 4.1 The rise of cluster‐level FWE

Cluster‐level FWE replaced voxel‐level FDR in 2016 and since has been the most popular inference method. It could be related to the publication of the seminal paper by Eickhoff et al. in that year, which through computation models strongly advocated the use of the former as the most appropriate method and the latter as a method to “be avoided” (Eickhoff et al., 2016). Until the date of writing this report, this paper has already been cited about 170 times. Also in 2016 the version of GingerALE 2.3.6 was released and it designated cluster‐level FWE as the default. Accompanying this was a technical report first published online in 2016 that described the errors inflating false positives in previous versions and fixed in version 2.3.6 (Eickhoff et al., 2017). This paper has been cited 81 times. It propelled researchers to switch to 2.3.6 or newer versions, consistent to our observations. A reporting guideline published by key opinion leaders in 2018 further credited the use of cluster‐level FWE for ALE meta‐analysis (Müller et al., 2018). Results from the current report suggested that all these movements successfully drove the community toward the use of cluster‐level FWE inference, consistent to a recent empirical assessment of task‐fMRI studies (Yeung et al., 2020).

### 4.2 Lack of analytic details and data for replication

Though there was an increasing trend in applying data redundancy filter (merging multiple Experiments from the same set of subjects into one, or selecting the most representative Experiment), these studies only accounted for 1/8 of all surveyed ALE papers. Treating dependent Experiments as independent might pose a potential bias from within‐group effects, which might significantly influence the final meta‐analytic outcome (Turkeltaub et al., 2012). Readers cannot easily probe into this issue because only 3.2% of surveyed ALE papers provided input files readily to be analysed. Most of the ALE papers only listed the included studies in a table, perhaps with the sample size (*n*) and number of foci. The variability/flexibility in the analytic pipeline of neuroimaging data (and undisclosed methodological details) has been constantly argued as a problem leading to reproducibility issues (Botvinik‐Nezer et al., 2020; Carp, 2012a, 2012b; Hong et al., 2019). Also, the lack of double‐checking on the accuracy of the coordinates entered into the analysis might render the results erroneous. Surely the context of successful replication needs to be defined on a case‐by‐case basis, ranging from the scale of macroscopic anatomical landmarks (e.g., insula) to some precise voxels or patterns of activation (Hong et al., 2019). In ALE meta‐analysis via GingerALE, the pipeline is relatively rigid besides the compilation of the input files. Researchers can conduct a replication of ALE meta‐analysis via Sleuth and GingerALE easily if the original authors have supplied a “Complete Citation” to the BrainMap database. This cannot be emphasized enough, as providing coded data and analytic pipeline to the BrainMap community can promote replication and extension of existing meta‐analyses (Fox et al., 2005). Meanwhile, some ALE papers provided a list of all source papers without complete bibliographic details, DOIs, or with broken links, so that we were unable to trace if these source papers were indeed indexed in the BrainMap database or not.

On the bright side, the neuroimaging community actually largely supported and contributed to the content of the BrainMap project as external contributors coded 61% of the 4826 source papers added to the BrainMap database. It is understandable that the authors seldom mentioned their contributions explicitly in their ALE papers, as the contribution is not directly related to the research methods and results, and the contribution can be done post publication. Therefore, we urge researchers to code all papers with Scribe, submit them to BrainMap, and state this in the Data Availability paragraph to inform readers as a formal documentation. BrainMap workspace and GingerALE input file should be provided as supplementary materials and all these files should be provided to BrainMap.org/pubs linked to the paper to make it a “complete citation.” To further enhance discoverability, the output ALE maps in nifti format should be uploaded to online repositories such as Archive of Neuroimaging Meta‐Analyses (ANIMA, https://anima.fz-juelich.de).

### 4.3 The need for data and code sharing transparency

Many journals nowadays request Data and Code Availability Statements as components separated from the main text, for instance, journals from Public Library of Science (PLOS), Springer Nature, BMC, and member journals of International Committee of Medical Journal Editors (ICMJE) (Hrynaszkiewicz, 2019). This practice is highly favourable, as it attracts the attention from the authors and the readers, so that everyone knows exactly where the information should be.

For Data Availability Statement, standard wording is suggested as follows:

“All data used in this manuscript for coordinate‐based meta‐analysis are available at www.brainmap.org. Published reports included in this meta‐analysis that were not available in BrainMap at the outset of the study were coded and submitted during the course of the study. The BrainMap workspace used for statistical analysis is provided as supplemental data.”

For Code Availability Statement, standard wording can be:

“All software tools used in this manuscript for coordinate‐based meta‐analysis are available for download at www.brainmap.org.”

### 4.4 Sample size not increasing

Though the annual median numbers of Papers per ALE paper and Experiments per analysis seemed to be on the rise, the overall data showed no increasing trend. Given that a meta‐analysis should include at least 17–20 Experiments (Eickhoff et al., 2016; Müller et al., 2018), 30.3% of the surveyed ALE papers met this requirement, 20.9% of them failed to meet, and the rest 48.8% could not be determined due to a lack of information. Precisely, from the 309 papers with number of Experiments per analysis reported, 126 had <17.0 and 183 had >17.0. In other words, 40.8% of the 309 papers failed to meet the requirement. The better‐powered studies were more likely to use cluster‐level FWE than less‐powered ones, but not clearly explain how to apply data redundancy filter or explicitly exclude ROI papers. One potential reason to include ROI papers might be a lack of original papers (Experiments). However, an actual ALE meta‐analytic report on phobia studies has demonstrated completely different results generated from whole‐brain studies versus a combination of ROI studies with them (Gentili et al., 2019). Readers should be aware that many ALE papers presented multiple meta‐analyses, so that some analyses might involve >17 Experiments whereas the rest might not. One possible way to evaluate the result robustness was to compute the Fail‐Safe N (FSN) by determining the number of noise studies that can be added (Acar et al., 2018; Gray et al., 2020). Perhaps researchers could consider explicitly labelling meta‐analyses with insufficient Experiments as being post hoc, to provide more interpretative detail following an “omnibus” style meta‐analysis with a large number of Experiments pooled together.

### 4.5 The need for meta‐analysis

As mentioned in the Section 1, one crucial motivation for conducting CBMA is to determine which findings in the literature are consistent across studies. False positives in neuroimaging can be attributed to multiple factors, such as small or heterogeneous samples, undisclosed flexibility in statistical testing, and suboptimal correction for multiple comparisons (Botvinik‐Nezer et al., 2020; Button et al., 2013; Carp, 2012a; Hong et al., 2019). By testing for spatial convergence across the findings from multiple neuroimaging studies, CBMA offers a practical solution to this problem (Gray et al., 2020). To the best of the authors' knowledge, this was the first study that surveyed the reporting quality of the overall meta‐analytic literature. Regarding sample size, previous studies focused on original studies only. For instance, it was found that in neuroimaging sample size increased by 0.74 per year between 1993 and 2010 and the median reached about 20 in 2012/2013 (Szucs & Ioannidis, 2020), 28.5 in 2015 (Poldrack et al., 2017) and 33 in 2017 (Yeung, 2018). Meanwhile, there was a range of advocated sample size for a group of subjects, such as 24 (Desmond & Glover, 2002), 40 (Geuter et al., 2018) or 100 (Turner et al., 2018). Of course, the required sample size depends on many parameters, for example, to replicate voxelwise or small areas of activation, a larger sample size is needed compared with a focus on clusters of voxels (Bossier et al., 2020). However, only 3%–4% of papers published in 2017/2018 had clear a priori power calculations (Szucs & Ioannidis, 2020). Therefore, some significant neuroimaging results from underpowered studies might be false positives. A well‐balanced meta‐analysis that pools data across all relevant studies may clear the doubts. Unfortunately, we found that only 30.3% of the surveyed ALE papers had an average of >17 Experiments per analysis as recommended by (Eickhoff et al., 2016; Müller et al., 2018). ALE papers that merely reported the number of original studies should also report the number of Experiments for each analysis. Otherwise, it defeated the purpose of increasing power by performing a meta‐analysis.

### 4.6 Future directions

CBMA has been applied most widely to group‐averaged, task‐control contrasts in healthy controls, which are coded and shared in the BrainMap database Task Activation (TA) sector. The present paper addresses solely TA‐sector data. Reports of ALE application to voxel‐based morphometric (VBM) data, however, are growing in number. VBM studies report groupwise, case–control contrasts comparing a disorder (e.g., Huntington's Disease) to a control group. VBM data are shared in the BrainMap VBM Sector (Vanasse et al., 2018). Application of ALE to voxel‐based physiological (VBP) data is a new development (Gray et al., 2020; Sha et al., 2018). VBP ALE discovers disease‐related alterations in hemodynamics, metabolism and neurovascular coupling by group‐averaged, case–control contrasts, in a strategy very similar to VBP ALE. A BrainMap VBP Sector is under construction by the BrainMap Team and will be released soon. When applying ALE to VBM or VBP data, compliance with the reporting standards recommended here is no less important.

Multivariate CBMA is another area of steady growth in the field, for which reporting standards are not yet well described and which was not addressed in the above‐described analyses. Multivariate CBMA is used to discover brain network properties, both structural and functional, that is, meta‐connectomics. Although meta‐connectomic methods use the same input data (standardized coordinates from published reports), the analyses applied be ALE‐based such as meta‐analytic connectivity modelling (MACM; Robinson et al., 2010) or use other network‐discovery approaches such as independent components analysis (ICA; Smith et al., 2009) or graph theory modelling (GTM; Crossley et al., 2014). Analysis and reporting standards for meta‐connectomic methods are still in evolution. To address this, the BrainMap Team has developed a BrainMap Community Portal (portal.brainmap.org) hosted by the Texas Advanced Computing Center (www.tacc.utexas.edu). The BrainMap Portal hosts all three BrainMap data sectors (TA, VBM, VBP) and the BrainMap software suite in a high‐performance computing environment. The portal also provides containerized meta‐connectomic applications (MACM, ICA, GTM) and a venue for sharing workspaces, algorithms and pipelines. The intention is to promote scientific transparency, rigor and reproducibility by openly sharing access to source data, algorithms and results for CBMA.

## 5 CONCLUSION

Through the survey of 603 ALE papers published in the 2010s, the following conclusions could be made:

1. Reporting standard: To embrace open science and adhere to PRISMA statement, the provision of Data and Code availability statements and flow chart of literature screening process should be much more encouraged. Cluster‐level FWE became the mainstream in recent years, but more ALE papers should exclude ROI papers and report details on data redundancy filter.
2. Study characteristics: the average number of Experiments per ALE meta‐analysis was 42.0 without a significant trend. About 30% of ALE papers met the requirement of >17 Experiments per analysis. These better‐powered studies were more likely to use cluster‐level FWE than less powered ones, but not on data redundancy filter or explicit exclusion of ROI papers. BrainMap database was queried as a data source by 77 of the 603 ALE papers, and by all of another 254 papers dealing with multi‐variate modelling, making up a total proportion of 331/857 (38.6%).
3. Data sharing: External contributors coded 61% of the 4826 source papers added into the BrainMap database during the period of 2010–2019. This ratio could only be demonstrated using data obtained by the BrainMap team but not from the content of the published papers, because the authors seldom mentioned their contributions to the BrainMap database explicitly.
4. See Box 1 for a summary of the recommendations.

## CONFLICT OF INTEREST

The authors have no relevant financial or non‐financial interests to disclose.

## ETHICS STATEMENT

This work did not involve human or animal subjects and hence ethical approval was not applicable.

## Data Availability

Data is available at Figshare (https://doi.org/10.6084/m9.figshare.21580998). This work did not use codes or scripts. Data was processed by Excel (Version 16.64 for Mac) and SPSS (Version 26.0 for Mac).
